# Smoldering multiple myeloma in transition: redefining early myeloma in the modern era

**DOI:** 10.1038/s41375-026-02979-2

**Published:** 2026-05-05

**Authors:** Ola Landgren

**Affiliations:** Sylvester Myeloma Institute, Sylvester Comprehensive Cancer Center, University of Miami, Miami, FL USA

**Keywords:** Translational research, Therapeutics, Drug development

## Abstract

Smoldering multiple myeloma (SMM) has long been viewed as an intermediate precursor state, monitored until symptoms or organ dysfunction signaled the transition to active multiple myeloma (MM). Over the past decade, however, major advances in molecular biology, modern imaging, and immunotherapy have reshaped our understanding of early plasma cell disorders. The recent FDA approval of daratumumab for high-risk SMM marks a historic turning point. For the first time, early intervention is not only feasible but clinically validated. This evolving framework challenges long-standing assumptions regarding diagnostic boundaries, risk stratification, and the traditional “watch-and-wait” paradigm. It is logical to conjecture that emerging and future technologies will redefine portions of today’s SMM population as early myeloma, while others may ultimately be reclassified as benign monoclonal gammopathies. An interesting question is whether there will be any SMM patients left? This article reviews how the field has arrived at this new therapeutic era, outlines its limitations, and highlights key scientific and clinical questions that will shape the next generation of SMM research and care.

## Introduction

For decades, SMM occupied a middle ground in hematology; biologically aligned with MM, yet clinically silent [1]. Patients were monitored but not treated, based on the belief that therapy prior to organ damage offered no survival advantage and carried unnecessary toxicity [2]. This doctrine remained largely unchallenged until immunomodulatory drugs, proteasome inhibitors, and monoclonal antibodies transformed outcomes for symptomatic disease [3–6].

**Fig. 1:**
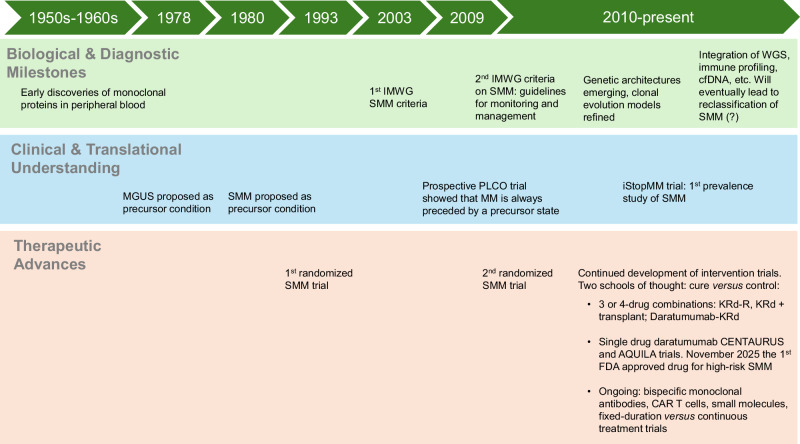
Evolution of biological and translational milestones as well as therapeutic advances in smoldering multiple myeloma.

The approval of daratumumab for high-risk SMM in 2025 represents a key moment in early MM clinical management [3, 5]. For the first time, early intervention has entered mainstream clinical practice, compelling a re-examination of how SMM is defined, stratified, and managed. The evolution of SMM – from a passive observation state to an actionable disease stage – reflects a broader shift toward detection and proactive treatment of early malignant clones (Fig. 1) [2–13].

## Historical evolution and shifting diagnostic boundaries

The term “smoldering” first appeared hematology in the 1960s when Rheingold and colleagues described smoldering acute leukemia, a slowly evolving form of the disease [14]. Inspired by Rheingold and colleagues, Kyle and Greipp applied the idea to multiple myeloma. In 1980, they identified six patients who met laboratory criteria for myeloma (≥10% marrow plasma cells or ≥3 g/dL M-protein) but who remained asymptomatic for more than five years [10]. They proposed the term SMM – a condition biologically aligned with MM, but clinically indolent.

Kyle and Greipp cautioned against treating these patients, reasoning that available therapies (i.e., primarily alkylating agents) were toxic and offered little long-term benefit [10]. They argued that observation was safer and spared patients from unnecessary anxiety and side effects. This “watch and wait” philosophy would dominate SMM management for decades.

In 2003, the introduction of formal SMM diagnostic criteria established a framework for research and clinical care, but it also codified heterogeneity, grouping biologically diverse patients into a single observational category. In parallel, as increasingly sensitive laboratory and imaging technologies have emerged [1], the borders between “precursor” and “malignancy” have become progressively more dynamic (Fig. 1) [2–13].

## Risk stratification: a foundation ready for reinvention

Risk stratification remains critically important to the management of patients with SMM, identifying individuals most likely to progress to MM. Based on work developed and promoted by the Mayo Clinic and others, the *so-called* “20/2/20 model” (based on serum M-protein ≥2 g/dL, free light chain ratio ≥20, and bone marrow plasma cells ≥20%) has gained widespread adoption for its practicality [15]. It provides a simple clinical framework to enrich high-risk disease and has become embedded by Mayo Clinic in several clinical guidelines.

It should be mentioned that the 20/2/20 model has inherent limitations (Table 1). It is grounded in clinical data and laboratory features detectable by earlier-generation technologies and does not incorporate biological data (such as genomics or host immune status). Similarly, it does not account for refined imaging biomarkers or dynamic changes in clonal burden. As a result, significant biological heterogeneity remains hidden beneath the model’s simplicity.

**Table 1:** Comparison of current 20/2/20-based risk stratification with emerging biological tools expected to refine classification of smoldering multiple myeloma.

Domain	Current model (20/2/20)	Limitations	Emerging technologies	Expected impact
Tumor burden	M-protein ≥2 g/dL; BMPC ≥ 20%	No clonal kinetics	Single-cell sequencing, ctDNA quantification	Dynamic prediction of progression
Light chains	FLC ratio ≥20	Static ratio insufficient	FLC kinetics, heavy/light chain suppression	Improved progression prediction
Imaging	Conventional MRI	Low sensitivity to microfoci	Whole-body DWI MRI, PET/MRI, immuno-PET	Earlier identification of occult bone disease
Genetics	Not included	No capture of high-risk genomics	FISH for del17p, t(4;14), 1q gain, next generation sequencing (NGS), single-cell sequencing	“Biologically high-risk SMM” classification
Microenvironment	Not included	No immune context	Immune profiling, spatial transcriptomics	Identifies permissive microenvironment
Dynamics	Not assessed	Only baseline	Serial ctDNA, growth-rate modeling	Reclassification of active biology

Going forward, risk stratification will certainly become more granular. By integrating next-generation sequencing, ctDNA assays, single-cell immune profiling, advanced imaging and other approaches, the field will continue to advance. In fact, it seems logical to conjecture that future technologies eventually will split SMM into at least two biologically distinct entities [1]:

A. Early multiple myeloma, characterized by aggressive evolutionary dynamics warranting therapy; and
B. Benign monoclonal gammopathy, encompassing individuals with highly stable clonal behavior who may never require treatment.

## The first FDA-approved treatment for SMM: a new standard of care

The FDA approval of daratumumab monotherapy for high-risk SMM in November of 2025 was based on the phase II CENTAURUS trial [5] and the subsequent phase III AQUILA trial [3]. It marks the first regulatory endorsement of early intervention in this disease state. The AQUILA trial demonstrated a substantial reduction in progression risk compared with observation (demonstrated a 51% reduction in progression risk with daratumumab vs. observation), validating the concept that immunotherapy can meaningfully alter the natural history of SMM.

This milestone challenges long-standing assumptions, but also it raises important clinical questions:

- Should monotherapy be fixed-duration, or continuous? In the AQUILA trial, only a few percent of the patients treated with daratumumab monotherapy achieved a complete response (CR), and virtually no patients obtained measurable residial disease (MRD)-negativity. This raises the question whether daratumumab monotherapy may require prolonged (lifelong?) duration for patients with high-risk SMM?
- Is monotherapy sufficient? Combination strategies (e.g., daratumumab + teclistamab, or daratumumab + lenalidomide and dexamethasone) may produce deeper and more durable responses. For example, will a sustained MRD negativity, induced by highly effective antibody combinations, facilitate fixed-duration therapy? Could such approaches become a path toward a cure for high-risk SMM?
- How do we prevent overtreatment? Treating biologically indolent SMM risks unnecessary toxicity and potential long-term immune dysfunction.

Despite these and many other complexities, the approval of daratumumab represents a key step toward preventive oncology in MM and intervening before irreversible organ damage occurs.

## The potential end of “watch and wait”

The traditional recommendation of observation for SMM has relied on two pillars: (i) clinical risk prediction models with limited precision, and (ii) a historical lack of effective, tolerable early treatments. Both pillars are eroding rapidly. Immunotherapy-based combination treatment regimens can achieve deep responses with manageable toxicity, while newer technologies increasingly distinguish aggressive biology from slow-growing clones.

Clinically important, the abandonment of observation has solved many problems, but it has already created new issues. For example, it has prompted the creation a new clinical category: “relapsed/refractory SMM”. This is true because the progression criteria for high-risk SMM patients are not updated to reflect biological behavior of SMM patients who show (early) signs of progression while on therapy. For example, a high-risk SMM patient who is progressing on (or after completed) active therapy, but is not meeting the diagnostic criteria for MM, is currently in this gray area (i.e., relapsed/refractory SMM). It seems logical to propose that emerging biomarkers of progression, such as dynamic changes in circulating myeloma cells, ctDNA mutation burden, immune microenvironment signatures, and other types of markers should be used to justify re-treating patients earlier (i.e. changing therapy) by redefining them as active MM. The final solution for this dilemma remains to be agreed upon by experts in the field (Fig. 1) [2–13].

Overall, the challenge is balancing precision with prudence: intervening early enough to prevent organ damage while avoiding unnecessary lifelong therapy for patients with benign disease.

## Population-based screening: opportunities and uncertainties

The prevalence of monoclonal gammopathy of undetermined significance (MGUS) and SMM far exceeds that of MM, prompting interest in whether screening might improve outcomes. Studies such as the Icelandic iStopMM trial [16] have demonstrated the feasibility of population screening, uncovering high rates of previously unrecognized monoclonal gammopathies and enabling early identification of individuals with high-risk SMM [12]. However, population-level benefits remain unproven. Potential drawbacks include, for example:

- Overdiagnosis and medicalization of stable disease
- Psychological burden for patients
- Increased demand for healthcare systems
- Risk of overtreatment if early therapy becomes widely available

Screening may eventually benefit high-risk subgroups defined by genetics or family history of plasma cell disorders, but large-scale screening remains investigational. The results of ongoing trials will determine whether screening becomes a future public health strategy [16].

## Technological advances set to redefine SMM itself

The trajectory of SMM cannot be separated from the trajectory of technological innovation. Several emerging tools are poised to reshape how the field defines and manages SMM. Novel approaches point toward a future in which the definition of SMM depends less on static thresholds and more on dynamic, biologically informed models of clonal behavior. Below are a few perspectives on this topic:

1. *Ultra-Sensitive MRD Assays.* MRD assessment at 10^−6^ or 10^−7^ sensitivity allows detection of biologically relevant residual clones long before clinical progression. Applying MRD methodologies to SMM may identify individuals whose disease behavior aligns with active MM, supporting earlier treatment [1].
2. *Whole-Genome and Single-Cell Sequencing.* Deep sequencing can reveal high-risk driver mutations, clonal diversity, and evolutionary pressure. These insights may help distinguish stable versus progressive clones [1].
3. *Advanced Imaging.* Whole-body diffusion-weighted MRI and PET/MRI can detect focal lesions undetectable by conventional MRI, potentially redefining early bone involvement thresholds. Also, novel imaging modalities, such as target immuno-PET, may help and move the needle for the field of imaging in SMM and MM [17].
4. *Immune Microenvironment Profiling.* Patterns of immune exhaustion, T-cell dysfunction, and microenvironmental permissiveness may forecast disease evolution independent of tumor burden [18].

## Future directions

In my opinion, several converging themes will shape the next decade of SMM research and care:

1. *Refining Diagnostic Categories*. As stated above, SMM may eventually be divided into at least two biologically meaningful states:

- Early multiple myeloma (eligible for therapy), and
- Benign monoclonal gammopathy (requiring minimal monitoring).

2. An interesting question is whether there will be any SMM patients left? Perhaps, there will be a (small) group of patients which may be interested in randomized trials with (versus without) early intervention? The future will tell.
3. *Curative-Intent Prevention Strategies.* In April 2026, the first result from a small (N=20) phase 2 trial raised important but unresolved questions about the role of cilta-cel CAR-T cell therapy in patients with high-risk SMM [19]. While the finding that all of the 20 treated patients are MRD-negative at a median follow-up of 15.3 months is encouraging, it is far too early to infer a meaningful progression-free or overall survival benefit, especially given that existing, less toxic options already delay progression with modest survival advantage. Furthermore, the safety signal is harder to overlook, reflected in a 35% rate of significant neurological toxicity, including persistent symptoms in some patients,. This represents a substantial risk in a population where many individuals may never progress to MM. In addition to these early toxicities, it will be important to continue studying safety over time to ensure there are no other late side effects, which have been reported some patient in relapsed/refractory MM patients treated with cilta-cel. Importantly, it goes without saying that the risk-benefit balance for drug development in patients with precursor disease (i.e. SMM) is fundamentally different from the relapsed/refractory MM setting, where higher toxicity is more acceptable due to immediate disease threat. There are other ongoing immunotherapy clinical studies focusing on high-risk SMM. For example, fixed-duration antibody-based immunotherapy (e.g., daratumumab + teclistamab, or other combinations) may achieve deep, sustained MRD negativity and possibly (functional) cure in some patients. Based on available information from presentations at conferences, ongoing trials already suggest unprecedented response depths (MRD negativity 10^-6 in the vast majority of patients) and none of the patients developed any neurological toxicities.
4. *Avoiding “Relapsed/Refractory SMM”.* Updating diagnostic (and progression) criteria is essential to avoid patients progressing biologically after early intervention but remaining “ineligible” for MM therapies because they lack CRAB features.
5. *Treatment Guided by Biomarkers.* It seems logical to believe that surrogate endpoints, such as biochemical progression, or conversion from MRD negativity to MRD positivity, will be used in management and treatment of both SMM and MM in the future. A key missing piece in the puzzle is the lack of reliable, reproducible blood-based MRD tools. Once available, it is highly likely that such tests will be introduced in the standard of care setting.
6. *International Harmonization.* The field of medicine will continue to reflect a broad variety of opinions, and that is great for innovation and research avenues. At the same time, it is important that the field continues to organize data-driven workshops and other initiatives to address the needs for updated SMM classifications, risk models, and endpoints. Harmonized guidelines will be crucial as new biomarkers and technologies enter clinical practice. It will continue to allow us to compare results across studies, create novel ideas, and build new collaborations.

In summary, the FDA’s approval of daratumumab for high-risk SMM marks a clear turning point, shifting the field from reactive care to pre-emptive treatment (Fig. 1) [2–13]. For the first time, efforts to halt MM before clinical progression are part of standard practice. Whether this strategy will ultimately deliver (functional) cures or simply establish a more favorable disease plateau is not yet known. Still, the increased pace of innovation – from immunotherapy and small molecules to increasingly sensitive (and ultimately blood-based) MRD assays – suggests that the future for individuals with SMM (and MM) is more promising than ever! Table 2.

**Table 2:** Scientific and clinical challenges in SMM and corresponding opportunities that are expected to shape the next decade.

Challenge	Current barrier	Opportunity	Potential clinical impact
Diagnostic thresholds	Fixed definitions	Biology-based definitions	Avoid misclassification
Risk stratification	20/2/20 score being too crude	Multi-omics, AI	More accurate risk prediction
Re-treatment triggers	CRAB features required	Surrogate biomarkers	Earlier access to therapy
Therapy duration	Poorly defined for SMM	Fixed-duration immunotherapy	Lower toxicity, cost
Relapsed/Refractory SMM	Potential new category	Redefine via biomarkers	Ensure eligibility for therapies
Screening	Unproven benefit	Ongoing iStopMM study data	Identify high-risk subgroups
Economics/toxicity	Unknown effects of early treatment	“Short”, deep-remission regimens	Sustainable preventive strategies
